# The prevalence, comorbidities, influencing factors, and identifying factors of non-obese fatty liver disease

**DOI:** 10.3389/fmed.2022.1038475

**Published:** 2022-11-15

**Authors:** Ning Gao, Jiang Deng, Jinhai Wang, Zhihua Zhou, Cong Yao, Mimi Zhou, Xin Xing, Qian Wang, Xiaolan Lu, Haitao Shi

**Affiliations:** ^1^Department of Infectious Disease, The Second Affiliated Hospital of Xi’an Jiaotong University, Xi’an, China; ^2^Department of Gastroenterology, The Second Affiliated Hospital of Xi’an Jiaotong University, Xi’an, China; ^3^Department of Health Management, The Second Affiliated Hospital of Xi’an Jiaotong University, Xi’an, China; ^4^Department of Nursing, The Second Affiliated Hospital of Xi’an Jiaotong University, Xi’an, China; ^5^Department of Gastroenterology, Pudong Hospital, Fudan University, Shanghai, China

**Keywords:** non-obese fatty liver disease, prevalence, influencing factor, triglycerides, non-alcoholic fatty liver disease (NAFLD)

## Abstract

**Objective:**

To analyze the prevalence, associated comorbidities, influencing factors, and identifying factors of non-obese fatty liver disease and to provide a reference for its prevention and treatment.

**Materials and methods:**

Firstly, to screen data obtained from the physical examinations of individuals conducted in the Second Affiliated Hospital of Xi’an Jiaotong University in 2021, subjects with complete data of abdominal ultrasonography, body mass index, age and sex were selected to analyze the prevalence of fatty liver disease and non-obese fatty liver disease. Secondly, to screen non-obese subjects who had data for triglycerides, fasting blood glucose, and so on, to analyze the complications, influencing factors, and identifying factors of non-obese fatty liver disease.

**Results:**

The prevalence of fatty liver disease was 27.8% (18,416/66,221), including 33.9% (11,921/35,131) in males and 20.9% (6,495/31,090) in females, revealing that the prevalence was significantly higher in males than in females (*P* < 0.001). There were 40,673 non-obese subjects screened in total, and the prevalence of non-obese fatty liver disease was 13.0% (5,307/40,673). The prevalence of non-obese fatty liver disease was 13.3% (2,208/16,572) in males and 12.9% (3,099/24,101) in females; the difference was not statistically significant (*P* = 0.17). The serum triglycerides level was elevated in 54.2% of subjects with non-obese fatty liver disease, and this was the most common abnormal metabolic index accompanying the disease. Logistic regression analysis showed that gender, age, body mass index, blood pressure, alanine aminotransferase, aspartate aminotransferase, fasting blood glucose, triglycerides, total cholesterol, and serum uric acid were independent risk factors for non-obese fatty liver disease (*P* < 0.001). For triglycerides, the area under the receiver operating characteristic curve in predicting non-obese fatty liver disease was the greatest (0.806).

**Conclusion:**

The prevalence of fatty liver disease and non-obese fatty liver disease determined by the physical examination of individuals was high, and the triglycerides is likely to be useful for the extensive screening of non-obese fatty liver disease.

## Introduction

In recent years, the prevalence of non-alcoholic fatty liver disease (NAFLD) has increased significantly, becoming the most common liver disease in the world and affecting approximately one in four adults. This causes a great burden on public health and economies worldwide.

Non-alcoholic fatty liver disease (NAFLD) is closely related to obesity, type 2 diabetes, and other metabolic diseases, and the prevalence of NAFLD in obese patients can be as high as 90% (1, 2). The correlation between a high body mass index (BMI) and NAFLD is well-established. However, there have been few studies on non-obese fatty liver disease, and this lack of research is not conducive to the prevention and treatment of related diseases. Therefore, this study examined a large sample of data obtained from physical examinations to analyze the prevalence, associated comorbidities, independent influencing factors, and identifying factors of non-obese FLD in order to provide a reference for the future prevention and treatment of related diseases.

## Materials and methods

### Study population

Physical examination data of subjects obtained from January to December 2021 including gender, age, BMI, and abdominal ultrasound were imported into Microsoft Excel 2016. The data included a total of 66,221 cases, including 35,131 males and 31,090 females. This study was approved by the Ethics Committee of the Second Affiliated Hospital of Xi’an Jiaotong University. All subjects remain anonymous and their information is not publicly available.

### Methods

The comprehensive data of the 66,221 individuals including BMI, sex, age, and abdominal ultrasound collected in 2021 was selected to analyze the prevalence of FLD and non-obese FLD. The comprehensive data of 36,507 non-obese individuals including BMI, gender, age, abdominal ultrasound, systolic blood pressure (SBP), diastolic blood pressure (DBP), aspartate aminotransferase (AST), alanine aminotransferase (ALT), total bilirubin (TBIL), fasting blood glucose (FBG), triglyceride (TG), total cholesterol (TC), and blood uric acid (UA) were selected for subsequent analysis of the independent influencing and identifying factors of non-obese FLD. Receiver operating characteristic (ROC) curves were used to evaluate the diagnostic efficacy of meaningful indicators. In this study, *P* < 0.05 indicated a statistically significant difference.

Ultrasound was performed by experienced physicians to diagnose FLD. The diagnostic criteria were as follows: (1) the echo of liver parenchyma was fine and enhanced, showing a misty shape, and the posterior echo was attenuated; (2) the intrahepatic duct structure was not clear; (3) the comparison between the liver and kidney was positive, the echo of the liver was slightly higher than that of renal parenchyma under normal circumstances, and the echo of the liver was evidently enhanced; (4) the liver was enlarged, full in shape, and had blunt edges.

According to the Asian BMI diagnostic criteria, a BMI < 18.5 kg/m^2^ is considered emasculated, a 18.5 ≤ BMI < 22.9 kg/m^2^ is considered normal, a BMI ≥ 23 kg/m^2^ is considered overweight, and a BMI ≥ 25 kg/m^2^ is considered obese. Therefore, cases of FLD in which the subjects had a BMI < 25 kg/m^2^ were defined as cases of non-obese FLD in this study.

According to the standards set by the Second Affiliated Hospital of Xi’an Jiaotong University, the values of TC > 5.2 mmol/L, TG > 1.7 mmol/L, and FBG > 6.1 mmol/L indicated that these indexes were elevated.

### Statistical analysis

Counting data were expressed as ratios and the Chi-square exact test was used for comparison. Quantitative data with a normal distribution were expressed as the mean ± standard deviation and compared using the *t*-test. Non-normal quantitative data were described by the interquartile range and tested using the Mann-Whitney *U* test. Variables were included in binary Logistic regression to analyze independent influencing factors, and a ROC curve was used to evaluate the diagnostic efficacy of indicators. Differences were considered statistically significant at *P* < 0.05. IBM SPSS 23.0 (SPSS Inc., Armonk, NY, USA) was used for all statistical analyses.

## Results

### Prevalence of fatty liver disease and non-obese fatty liver disease determined by physical examinations conducted in 2021

The prevalence of FLD in individuals of the Second Affiliated Hospital of Xi’an Jiaotong University in 2021 was 27.8% (18,416/66,221), including 33.9% (11,921/35,131) in males and 20.9% (6,495/31,090) in females. The prevalence of FLD was significantly higher in males than females (*P* < 0.001). The prevalence of non-obese FLD in all non-obese subjects was 13.0% (5,307/40,673), including 13.3% (2,208/16,572) in males and 12.9% (3,099/24,101) in females, indicating no significant difference in the prevalence of non-obese FLD between males and females (*P* = 0.17).

### Prevalence of fatty liver disease after body mass index stratification of physical examinations conducted in 2021

After stratification by BMI, the prevalence of FLD in the lean, normal, overweight, and obese subjects that had undergone a physical examination was 3.5%, 9.6%, 24.7%, and 51.3%, respectively, thereby showing an increase in FLD prevalence with increasing BMI. The difference between the four groups was statistically significant (*P* < 0.001) (Table 1). The prevalence of non-obese FLD was different between the different age groups (*P* < 0.001), and the prevalence of non-obese FLD was the highest in those aged > 60. The prevalence of non-obese FLD was different between males and females among the different age groups; in those aged above 60 years, the prevalence of non-obese FLD was significantly higher in females than in males (*P* < 0.001) (Table 2), which may be related to the decrease in estrogen level and accompanying increased risk of metabolic diseases experienced by females in this age cohort.

**TABLE 1:** Prevalence of fatty liver disease (FLD) after body mass index (BMI) stratification of physical examinations conducted in 2021.

BMI (kg/m^2^)	Total (%)	Male (%)	Female (%)	χ^2^	*P*
< 18.5	3.5(221/6333)	1.1(14/1282)	4.1(207/5051)	27.44	<0.001
18.5–22.9	9.6(2151/22438)	7.2(641/8885)	11.1(1510/13553)	95.49	<0.001
23–24.9	24.7(2935/11902)	24.2(1553/6405)	25.1(1382/5497)	1.27	0.259
≥ 25	51.3(13109/25548)	52.3(9713/18559)	48.6(3396/6989)	28.50	<0.001

BMI, body mass index.

**TABLE 2:** Prevalence of non-obese fatty liver disease (FLD) among non-obese physical examinees of different ages.

Age (years)	Total (%)	Male (%)	Female (%)	χ^2^	*P*
< 30	5.7(465/8178)	5.6(204/3632)	5.7(261/4546)	0.06	0.809
30–39	10.0(1319/13182)	11.6(626/5386)	8.9(693/7796)	26.43	<0.001
40–49	15.2(1170/7709)	18.6(557/2987)	13.0(613/4722)	45.62	<0.001
50–59	20.2(1333/6604)	18.9(501/2647)	21.0(832/3957)	4.34	0.037
> 60	20.4(1020/5000)	16.7(320/1920)	22.7(700/3080)	26.75	<0.001

### Comparison of baseline data between non-fatty liver disease and fatty liver disease subjects in non-obese subjects examined in 2021

A total of 36,507 non-obese physical examinees with complete data on their sex, age, SBP, DBP, ALT, AST, FBG, TG, TC, and UA were screened for multivariate analysis. Among the non-obese physical examiners, there were statistically significant differences found for gender, age, SBP, DBP, ALT, AST, FBG, TG, TC, and UA between the non-obese FLD group and the non-FLD group (*P* < 0.05). Compared to non-obese subjects without FLD, patients with non-obese FLD had higher age, BMI, SBP, DBP, ALT, AST, FBG, TG, TC, and UA (Table 3).

**TABLE 3:** Comparison of baseline data between non-fatty liver disease (FLD) and FLD subjects in non-obese subjects examined in 2021.

Characteristics	Non-FLD (*n* = 31,757)	Non-obese FLD (*n* = 4,750)	Statistical value	*P*
male/female	13824/17933	2140/2610	χ^2^ = 3.89	0.049
Age (years)	41.0 ± 13.7	46.5 ± 13.2	*t* = 26.87	<0.001
BMI (kg/m^2^)	21.1 ± 2.5	22.8 ± 1.9	*t* = 58.04	<0.001
FBG (mmol/L)	5.14 ± 0.87	5.76 ± 1.56	*t* = 26.44	<0.001
SBP (mmHg)	118.6 ± 14.2	127.3 ± 15.2	*t* = 36.88	<0.001
DBP (mmHg)	75.0 ± 9.5	80.8 ± 9.8	*t* = 38.25	<0.001
ALT (U/L)	17.0 (14.0–21.0)	22.0 (17.0–29.0)	*Z* = 47.12	<0.001
AST (U/L)	16.0 (12.0–21.0)	22.0 (17.0–30.0)	*Z* = 48.60	<0.001
TC (mmol/L)	4.32 ± 0.83	4.69 ± 0.93	*t* = 25.92	<0.001
TG (mmol/L)	0.99 (0.73–1.37)	1.80 (1.30–2.52)	*Z* = 68.14	<0.001
UA (μmol/L)	287.2 ± 73.8	348.0 ± 81.0	*t* = 48.78	<0.001

BMI, body mass index; FBG, fasting blood glucose; SBP, systolic blood pressure; DBP, diastolic blood pressure; ALT, alanine transaminase; AST, aspartate aminotransferase; TBIL, total bilirubin; TC, total cholesterol; TG, triglycerides; UA, uric acid.

### Analysis of metabolic abnormalities in non-obese fatty liver disease subjects in 2021

Of the 4,750 non-obese FLD subjects, 2,140 were male, of whom, 452 had blood uric acid levels > 420 μmol/L; 2,610 were female, of whom, 975 had a blood uric acid level > 360 μmol/L. A total of 1,247 (26.3%) of the non-obese FLD subjects had serum TC > 5.2 mmol/L; 872 subjects (18.4%) had FBG > 6.1 mmol/L; 2,573 subjects (54.2%) had TG > 1.7 mmol/L. Elevated serum TG was found to be the most common abnormal metabolic index in subjects with non-obese FLD.

### Logistic regression analysis of non-obese fatty liver disease

The variables included for analysis included gender, age, BMI, SBP, DBP, ALT, AST, FBG, TG, TC, and UA. After adjusting for gender, age, and other factors, binary logistic regression analysis determined that the independent influencing factors of non-obese FLD in non-obese subjects were gender, age, BMI, SBP, DBP, ALT, AST, FBG, TG, TC, and UA (*P* < 0.001) (Table 4).

**TABLE 4:** Logistic regression analysis of non-obese fatty liver disease (FLD).

Characteristics	B	*P*	OR	95% CI for OR
Male/female	0.329	< 0.001	1.390	1.290, 1.496
Age (years)	0.007	< 0.001	1.007	1.004, 1.010
BMI (kg/m^2^)	0.241	< 0.001	1.273	1.248, 1.298
FBG (mmol/L)	0.006	< 0.001	1.006	1.003, 1.010
SBP (mmHg)	0.018	< 0.001	1.018	1.013, 1.023
DBP (mmHg)	0.223	< 0.001	1.250	1.215, 1.285
ALT (U/L)	0.009	< 0.001	1.009	1.005, 1.012
AST (U/L)	0.016	< 0.001	1.016	1.013, 1.019
TC (mmol/L)	0.081	< 0.001	1.084	1.040, 1.130
TG (mmol/L)	0.644	< 0.001	1.903	1.826, 1.984
UA (μmol/L)	0.005	< 0.001	1.005	1.005, 1.006
Constant	–14.523	< 0.001	–	

BMI, body mass index; FBG, fasting blood glucose; SBP, systolic blood pressure; DBP, diastolic blood pressure; ALT, alanine transaminase; AST, aspartate aminotransferase; TC, total cholesterol; TG, triglycerides; UA, uric acid.

### Receiver operating characteristic to evaluate the diagnostic efficacy of each index in diagnosing non-obese fatty liver disease

**FIGURE 1:**
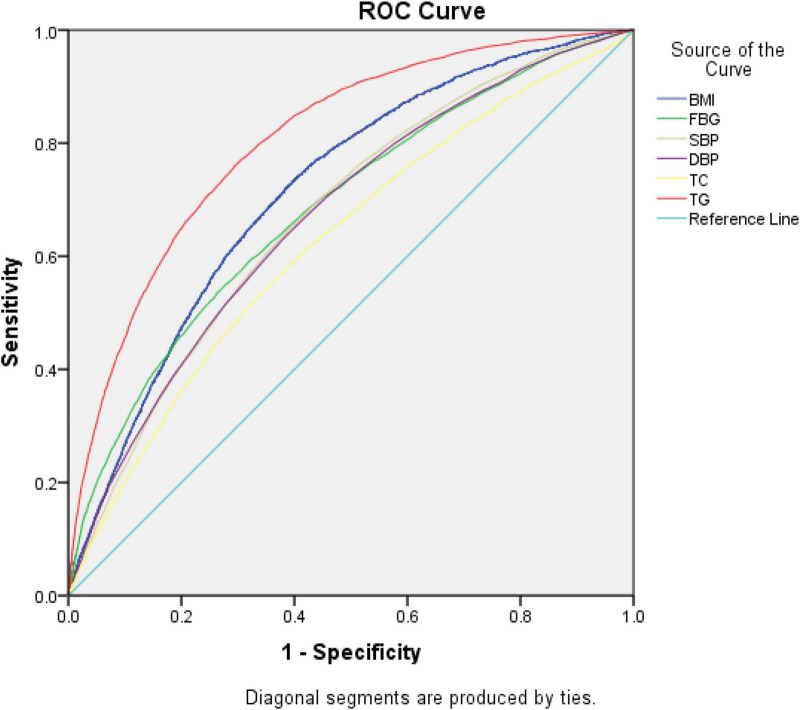
Receiver operating characteristic (ROC) curve for predicting the occurrence of non-obese fatty liver disease.

Taking BMI, FBG, SBP, DBP, TC, and TG as the test variables, we constructed a ROC chart and calculated the AUROC (Figure 1 and Table 5). The results showed that the AUROC value of TG was 0.806, which was greater than that of all the other variables, and the diagnostic efficacy of TG was superior among all variables. Taking the value corresponding to the maximum Youden index as the cutoff value (1.29), the specificity was 70.6% and the sensitivity was 76%.

**TABLE 5:** Receiver operating characteristic (ROC) curve evaluation of the diagnostic efficacy of each index in diagnosing non-obese fatty liver disease (FLD).

Variable	Area	*Std*	*P*	95% CI for OR	Cut-off point	Sensitivity	Specificity
BMI (kg/m^2^)	0.716	0.004	< 0.001	0.709, 0.724	22.0	0.741	0.595
FBG (mmol/L)	0.685	0.004	< 0.001	0.677, 0.694	5.31	0.541	0.731
SBP (mmHg)	0.672	0.004	< 0.001	0.664, 0.680	120.5	0.648	0.609
DBP (mmHg)	0.669	0.004	< 0.001	0.661, 0.677	77.5	0.633	0.618
TC (mmol/L)	0.625	0.004	< 0.001	0.616, 0.633	4.56	0.543	0.651
TG (mmol/L)	0.806	0.003	< 0.001	0.80, 0.812	1.29	0.76	0.706

BMI, body mass index; FBG, fasting blood glucose; SBP, systolic blood pressure; DBP, diastolic blood pressure; TC, total cholesterol; TG, triglycerides.

## Discussion

With modern improvements in material living standards and an increase in the cultural pressure to work long hours, the prevalence of FLD has increased significantly, becoming the most common liver disease in the world (3–5). Our results show that the prevalence of FLD determined by physical examinations conducted in 2021 was 27.8% for both sexes. In 2016, a meta-analysis of 8,515,431 individuals showed that the worldwide prevalence of NAFLD in adults was 6.3–45% (25.2%, 95% CI: 22.1–28.7%). The highest prevalence was found in the Middle East at 31.8%, while Africa had the lowest prevalence at 13.5% (4). The prevalence of NAFLD in China increased from 25.4% in 2008–2010 to 32.3% in 2015–2018 (6), while the prevalence within China was found to differ among regions; Taiwan had the highest incidence at 39.9%, Northwest China had a prevalence of 33.8%, and Hong Kong had a prevalence of 31.5%. This variation among populations may be related to differences in social and environmental factors such as the economy, air pollution, and eating habits (6).

In a 14-country meta-analysis, the global prevalence of lean NAFLD was found to be 4.1% (95% CI: 3.4–4.8%) in the overall population and 9.7% (95% CI: 7.7–11.8%) in the lean population (7), while the present study showed that the prevalence of non-obese FLD was 13.0% (5,307/40,673). In 2020, a meta-analysis examining 24 countries and regions showed that among the NAFLD population, 19.2% (95% CI: 15.9–23.0%) were thin, while 40.8% (95% CI: 36.6–45.1%) were non-obese. In the general population, 12.1% (95% CI: 9.3–15.6%) had non-obese NAFLD, while 5.1% (95% CI: 3.7–7.0%) had lean NAFLD (8). Different studies may obtain different findings due to both different diagnostic criteria and differences in weight category standards set for different global populations. Differences may also be related to factors such as regional factors, ancestry, and gene polymorphisms (9–12). Among the general population, the prevalence of lean NAFLD was found to be the highest in Asia (4.8%), followed by Oceania (3.5%), North America (3.1%), and Europe (2.2%).

This study revealed that the prevalence of FLD in emaciated, normal BMI, overweight, and obese people was 3.5%, 9.6%, 24.7%, and 51.3%, respectively. The prevalence of FLD increased with increasing BMI, and high BMI was determined to be an independent risk factor for FLD. It is worth noting that even for non-obese subjects, increased BMI is still an independent risk factor for non-obese FLD. This study shows that the risk of non-obese FLD increases by 0.3 times for each unit of increase in BMI. Previous studies have pointed out that there are two subtypes of lean NAFLD: one is characterized by the presence of visceral fat and insulin resistance, and another is characterized by a normal BMI with liver steatosis secondary to monogenic disease (10). Many Asians with NAFLD are non-obese, and these individuals have a significantly increased risk of cardiovascular disease and diabetes, even among those with a normal BMI (13).

Studies have shown that visceral obesity increases the risk of NAFLD in non-obese people, thereby functioning as a risk factor that is independent of BMI and subcutaneous fat. Subcutaneous fat and BMI are associated with moderate to severe NAFLD, while visceral fat is associated with mild to severe NAFLD. The waist-to-hip ratio has a superior ability to screen for abdominal obesity compared to BMI (14). Therefore, FLD cannot be screened based on BMI alone. Some non-obese physical examinees may be affected by accompanying abnormal conditions such as uneven body fat distribution, high TG, and excessively rapid rates of weight loss. For such cases involving excessively rapid weight loss, although weight is reduced, a large amount of fat is consequently decomposed, thereby exceeding the metabolism and transport capacity of the liver. Excessive TG then accumulates in the liver to contribute to the development of non-obese FLD. After analyzing the metabolic indexes of non-obese FLD subjects, this study found that 54.2% of non-obese FLD subjects had elevated TG, and this represented the most common abnormal metabolic index of non-obese FLD, which was consistent with previous relevant research results (15).

This study revealed that gender, age, SBP, DBP, ALT, AST, FBG, TG, TC, and UA are independent factors affecting the occurrence of non-obese FLD in non-obese physical examinees, which is consistent with previous research findings (8, 15–17). NAFLD is a manifestation of metabolic syndrome involving the liver, and metabolic abnormalities increase the risk of NAFLD. Studies have shown that compared to NAFLD subjects characterized by a high BMI, the incidences of metabolic syndrome-related indicators and metabolic complications in thin NAFLD subjects are improved (7, 18, 19). However, the risk of metabolic disorders in lean NAFLD remains high (20–22). Both timely prevention and treatment are required for the successful treatment of NAFLD. We found that the most commonly abnormal index value in non-obese FLD subjects was the elevation of serum TG, whereby the corresponding area under the working characteristic curve for subjects was the greatest, which is consistent with the results of previous studies (15). Therefore, the TG level appears to be the best predictor for the initial screening of non-obese FLD from non-obese subjects. When combined with the ALT level (15), the diagnostic efficiency can be further improved.

This was a large, cross-sectional study based on a population undergoing physical examination, it is difficult to supplement such data with drinking history, glycosylated hemoglobin, and medication history, and this represents a deficiency of the current study (23–25). However, NAFLD has been renamed as metabolic associated fatty liver disease, whereby alcohol consumption is no longer emphasized and more attention is being paid to its associated metabolic diseases, such as hyperlipidemia and obesity. Investigating such metabolic abnormalities by measuring various metabolic indexes comprised one of the purposes of this study.

## Conclusion

This study elucidated the prevalence of both FLD and non-obese FLD among physical examinees that had attended the Second Affiliated Hospital of Xi’an Jiaotong University in 2021. The results reveal the prevalence of these diseases to be high and often complicated by glucose and lipid metabolism abnormalities. Effective measures should be taken promptly in such patients to actively prevent and reduce the damage caused by related diseases. Timely intervention can be improved by effective screening, and the index of serum triglycerides is likely to be a useful indicator for the extensive screening of non-obese FLD. This study was a single-center, large sample study, and so will need to be confirmed by a multi-center, prospective study.

## Data availability statement

The raw data supporting the conclusions of this article will be made available by the authors, without undue reservation.

## Ethics statement

The studies involving human participants were reviewed and approved by the Ethics Committee of the Second Affiliated Hospital of Xi’an Jiaotong University. Written informed consent from the patients/participants was not required for this study.

## Author contributions

JW, XL, and HS conceived and designed the study. JD, ZZ, CY, MZ, XX, and QW collected the data. JD analyzed the data. NG and JD wrote the manuscript. All authors have made a significant contribution to this manuscript and have approved the final manuscript.

## Abbreviations

ALT: alanine transaminase
AST: aspartate aminotransferase
BMI: body mass index
DBP: diastolic blood pressure
FBG: fasting blood
NAFLD: non-alcoholic fatty liver disease
ROC: receiver operating characteristic
SBP: systolic blood pressure
TC: total cholesterol
TBIL: total bilirubin
TG: triglycerides
UA: uric acid.
